# Supplementary material for: Fabrication of Metasurfaces on Building Construction Materials for Potential Electromagnetic Applications in the Microwave Band
Source: Materials (Basel). 2022 Oct 19;15(20):7315. doi: 10.3390/ma15207315 (PMC9611122; doi:10.3390/ma15207315)
Supplement: Supplementary file 1 [file materials-15-07315-s001.zip › materials-1906277-supplementary.pdf]

Article

Fabrication of Metasurfaces on Building Construction Materials for Potential Electromagnetic Applications in the Microwave Band

Zacharias Viskadourakis [1,*], Konstantinos Grammatikakis [2], Klytaimnistra Katsara [1,3], Argyri Drymiskianaki [2] and George Kenanakis [1]

[1] Institute of Electronic Structure and Laser (IESL), Foundation for Research and Technology—Hellas (FORTH), N. Plastira 100, Vassilika Vouton, 70013 Heraklion, Greece
[2] Materials Science and Technology Department, University of Crete, Vassilika Vouton, 70013 Heraklion, Greece
[3] Department of Agriculture, Hellenic Mediterranean University—Hellas, Greece, Estavromenos, GR-714 10 5 Heraklion, Greece
* Correspondence: zach@iesl.forth.gr; Tel.: +30-2810-391921

Abstract: Energy self-sufficiency, as well as optimal management of power in buildings is gaining importance, while obtaining power from traditional fossil energy sources is becoming more and more expensive. In this context, millimeter-scale metasurfaces can be employed to harvest energy from microwave sources. They can also be used as sensors in the microwave regime for efficient power management solutions. In the current study, a simple spray printing method is proposed to develop metasurfaces in construction materials, i.e., plasterboard and wood. Such materials are used in the interior design of buildings; therefore, the implementation of metasurfaces in large areas, such as walls, doors and floors, is realized. The fabricated metasurfaces were characterized regarding their electromagnetic performance. It is hereby shown that the investigated metasurfaces exhibit an efficient electromagnetic response in the frequency range (4–7 GHz), depending on the MS. Thus, spray-printed metasurfaces integrated on construction materials can potentially be used for electromagnetic applications, for buildings' power self-efficiency and management.

Keywords: metasurfaces; microwaves; energy harvesting; electromagnetic applications; construction materials; wood; plasterboard; power management

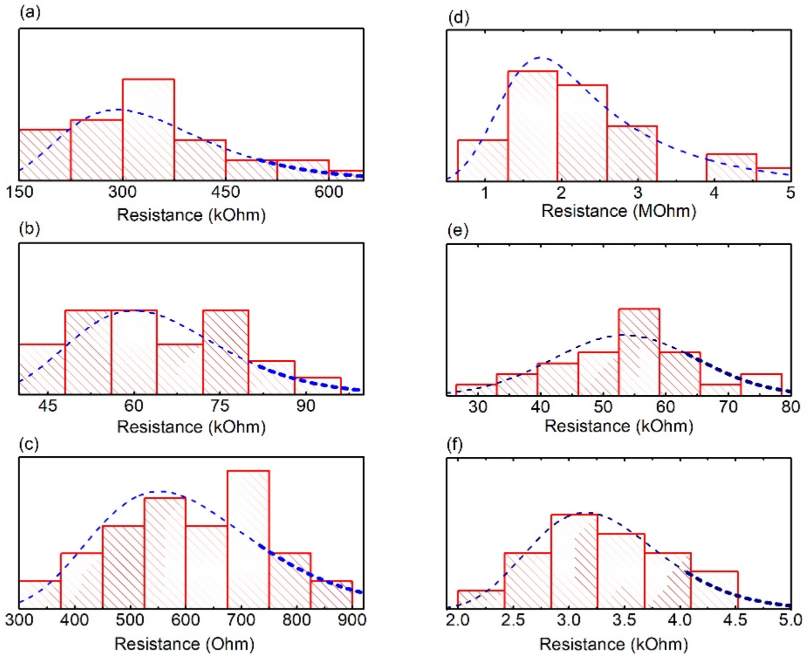

Figure S1. Resistance measurement of MSs printed on wood. Cut-wire MS, made of **(a)** graphite, **(b)** carbon paste and **(c)** HSF54. SRRs deposited, using **(d)** graphite, **(e)** carbon paste and **(f)** HSF54, respectively. Dash lines are guides to the eye.

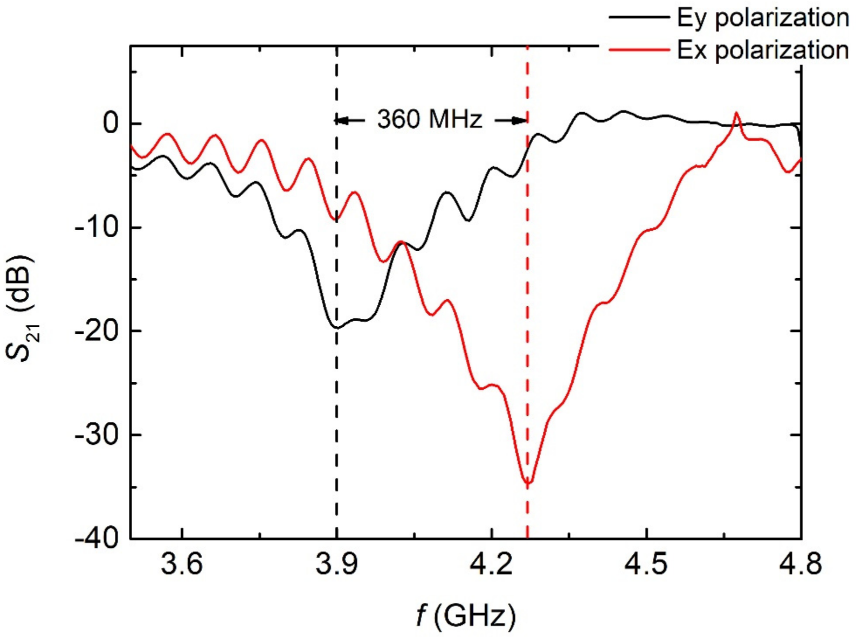

Figure S2. Electromagnetic response of the PCB-printed cut-wire metasurfaces, with respect to different poloarizations Ey (black line) and Ex (red line). A clear resonance shift is observed. .

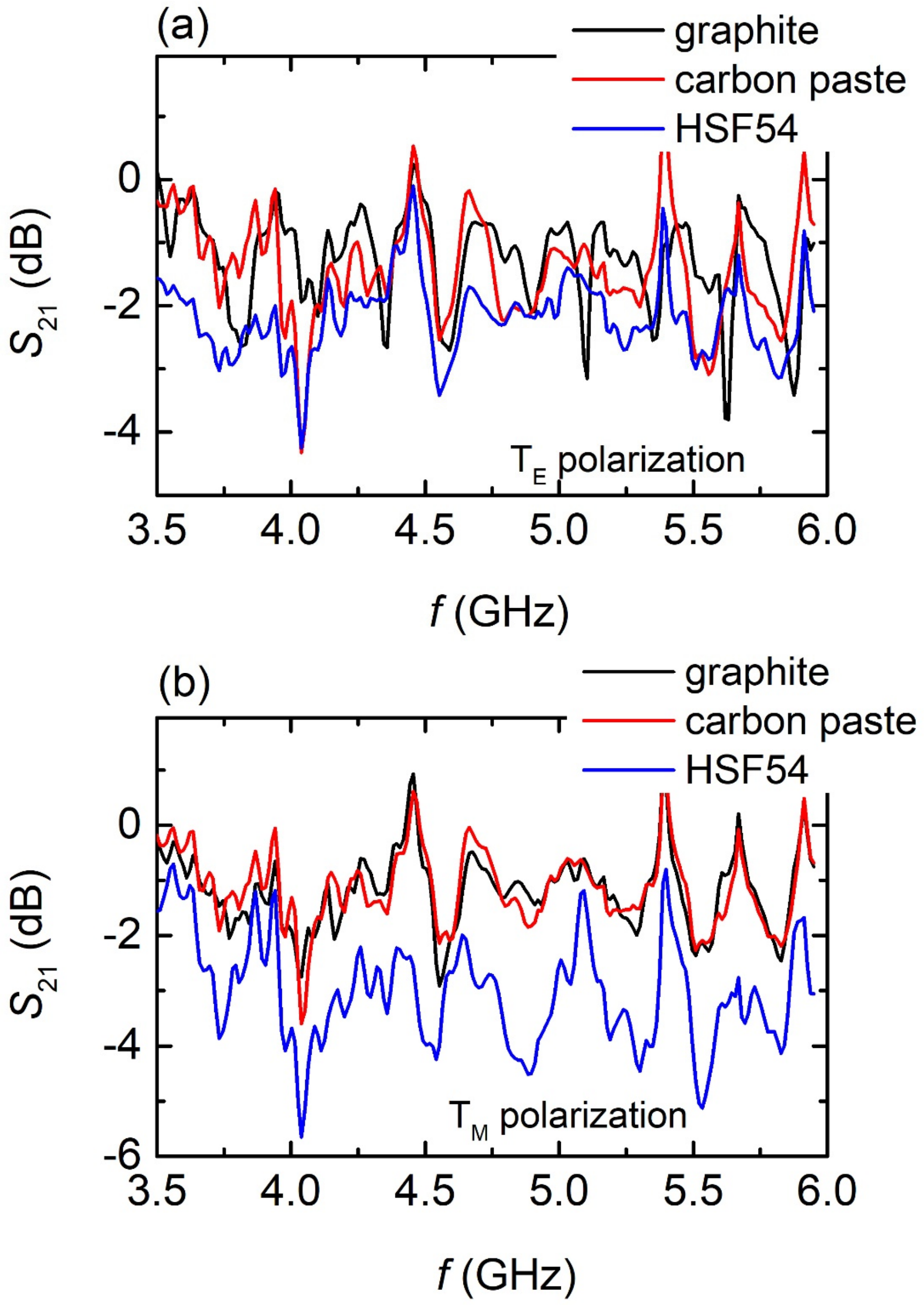

Figure S3. S_{21} vs. frequency for all SRRs printed on wood in the **(a)** T_E polarization and **(b)** T_M polarization.
